# Does universal health insurance coverage reduce unmet healthcare needs in China? Evidence from the National Health Service Survey

**DOI:** 10.1186/s12939-021-01385-7

**Published:** 2021-01-21

**Authors:** Shenping Zhou, Tianyu Huang, Anqi Li, Zhonghua Wang

**Affiliations:** 1 School of Health Policy & Management, Nanjing Medical University, 101Longmian Avenue, Jiangning District, Nanjing, 211166 P.R. China; 2 Creative Health Policy Research Group, Nanjing Medical University, Nanjing, 211166 China; 3 Center for Global Health, Nanjing Medical University, Nanjing, 211166 China

**Keywords:** Universal health insurance coverage, Unmet healthcare needs, Non-use of healthcare services, Inequality

## Abstract

**Background:**

China has nearly achieved universal health insurance coverage, but considerable unmet healthcare needs still exist. Although this topic has attracted great attention, there have been few studies examining the relationship between universal health insurance coverage and unmet healthcare needs. This study aimed to clarify the impact of universal health insurance coverage and other associated factors on Chinese residents’ unmet healthcare needs.

**Methods:**

Data was derived from the fourth, fifth, and sixth National Health Service Survey of Jiangsu Province, which were conducted in 2008, 2013, and 2018, respectively. Descriptive statistics were used to analyze the prevalence of unmet healthcare needs. Binary multivariate logistic regression was used to estimate the association between unmet healthcare needs and universal health insurance coverage, along with other socioeconomic factors.

**Results:**

8.99%, 1.37%, 53.37%, and 13.16% of the respondents in Jiangsu Province reported non-use of outpatient services, inpatient services, physical examinations, and early discharge from hospital, respectively. The trend in the prevalence of unmet healthcare needs showed a decline from 2008 to 2018. Health insurance had a significant reducing effect on non-use of outpatient services, inpatient services, or early discharge from hospital. People having health insurance in 2013 and 2018 were significantly less likely to report unmet healthcare needs compared to those in 2008. The effect of health insurance and its universal coverage on reducing unmet healthcare needs was greater in rural than in urban areas. Other socioeconomic factors, such as age, marital status, educational level, income level, or health status, also significantly affected unmet healthcare needs.

**Conclusions:**

Universal health insurance coverage has significantly reduced Chinese residents’ unmet healthcare needs. Policy efforts should pay more attention to the benefits of health insurances in rural areas and optimize urban-rural health resources to promote effective utilization of healthcare.

## Introduction

The World Health Organization defines universal health coverage (UHC) as the ability for all people to access equal health services that are effective and do not expose the user to financial hardship [1], and this is a major sustainable development goal all over the world. These health services include health promotion, prevention, treatment, rehabilitation, and palliative care [2]. Social health insurance can effectively reduce the medical burden and promote the utilization and accessibility of health services [3–5]. And the improvement of its coverage can effectively reduce the inequality of health service utilization caused by financial hardship, improve the utilization and accessibility of health services and reduce the degree of healthcare underutilization, so as to promote the realization of UHC [6, 7].With the implementation of the New Medical Reform Plan in 2009, China has established a social health insurance system framework consisting of Urban Employees Medical Insurance, Urban Residents’ Medical Insurance, and the New Rural Cooperative Medical Scheme, and these cover the employed urban population, unemployed urban residents, and rural residents, respectively. Outpatient services, inpatient services, critical illness mutual assistance, etc. are all included in the social health insurance. Health insurance coverage rate dramatically rose from 22% in 2003 to nearly 97% in 2019 [8]. China now has almost complete universal health insurance coverage, but there is still a dramatic disparity between universal health insurance coverage and universal health coverage [9].

Among the three systems, UEMI was first formally established in 1998, all the employed in urban areas should participate UEMI compulsorily and the health insurance premiums of UEMI are shared by both employers and employees. Followed by the release of “Decision of the CPC Central Committee and the State Council on Further Strengthening Rural Health Work” [10], NRCM was established in 2003. It is a health insurance system jointly funded by individuals, collectives and the government. Every local government has an escalating mandatory standard for improving coverage each year. Furthermore, in practice, if one family member was not insured, the whole family would be refused to participate in the insurance, therefore, NRCM is actually mandatory. URMI was established in 2007 and the health insurance premiums is mainly paid by families (individuals) and government subsidies. Although the original design of URMI is voluntary, it also has the binding problem——insured in family-unit, and the proportion of people directly participating in the insurance in individuals is very low, so it is partially mandatory indeed.

Equitable access to healthcare according to need, regardless of demographic, ability to pay, or social background is the main connotation of UHC [11]. Although a series of reforms to health insurance have been implemented since the New Medical Reform Plan in 2009, healthcare access in China remains highly unequal across different sub-populations and different areas [12].As a commonly used indicator of access to healthcare, unmet need is defined as the subjective perception of not receiving appropriate medical help when it is required [13–15]. Compared with healthcare utilization, the degree of healthcare underutilization can accurately reflect healthcare accessibility because healthcare utilization only reflect the actual use of health services by demanders(effective demand), which can not truly and concretely represent the whole healthcare demand [16–18]. Unmet healthcare needs are mainly a result of limited availability or unavailability of healthcare services, such as outpatient visits, hospitalization, or physical examinations, when they are needed [19]. People often choose not to use health services for reasons such as their cost, the travel distance required, their quality, the equipment available in medical institutions, or a lack of knowledge about health. This is one of the most important healthcare access issues that needs to be addressed, because unmet medical needs can not only result in a population having a poorer health status but can also lead to increasing health inequalities, thus affecting UHC [20].

Many studies have explored non-use of healthcare services and its associated factors. Several studies indicate an association between higher rates of non-use of healthcare services and female sex, younger age, rural living, lack of health insurance, lack of financial support, low levels of education, and poor health [12, 14, 21–26]. For example, Li et al. reported that social health insurance could significantly reduce foregone care in outpatient and inpatient situations [27]. Lucevic et al. confirmed that the most vulnerable groups at risk of unmet healthcare needs were women, those with only a primary or secondary education, people with poor health status, those living outside the capital, and those from the lowest income quintiles [15]. Sibley and Glazier found that unmet need was more common among women, younger people, those with higher educational attainment, and those with lower household income [10]. Ronksley et al. found that chronic conditions and distress were significantly associated with unmet healthcare needs [22]. However, most studies focusing on underutilization of healthcare have been limited to specific sub-populations or specific areas [28] or have focused on only one type of non-use of health services [26, 29–34]. With respect to those studies that have examined health insurance, most have focused on a certain kind of health insurance or were limited to cross-sectional data [35–39].

Although researchers and policymakers are highly interested in unmet healthcare needs, studies on the association between universal health insurance coverage and unmet healthcare needs are scarce in China. With the rapid improvement of social health insurance coverage after the New Medical Reform Plan 2009, it is imperative to explore the impact of the process of universal coverage of health insurance on health services underutilization. In this paper, we used data from the National Health Service Surveys in Jiangsu Province in China (2008–2018) to explore whether full health insurance coverage actually reduces unmet healthcare needs. The survey results represent pooled cross-sectional data that span the period of universal implementation of the basic health insurance system in China. We calculated the prevalence of healthcare underutilization, analyzed the main causes of unmet healthcare needs, and compared the prevalence of healthcare underutilization between urban and rural areas. Furthermore, we estimated the association between universal health insurance coverage, other socioeconomic factors, and unmet healthcare needs. The results of this study will provide scientific evidence for improving the social health insurance policy and improving the accessibility of health services, so as to achieve universal health coverage.

## Methods

### Data sources

The data came from the fourth, fifth, and sixth National Health Service Survey (NHSS) of Jiangsu Province in 2008, 2013, and 2018, respectively. The NHSS is a five-yearly survey administered by the Center for Health Statistics and Information of the National Health Commission. The survey adopted the method of multi-stage stratified random sampling, and a total of 156 counties (cities and districts) in 31 provinces were selected. Five sample towns (streets) were randomly selected for each sample county (cities or districts), two sample villages (neighborhood committees) were randomly selected for each sample town (street), and 60 households were randomly selected for each sample village (neighborhood committee), examining a total of 93,600 households (a population of nearly 300,000 people). Jiangsu Province is a densely populated area located in the east of mainland of China and has a population of 80.293 million. Nineteen counties were sampled in the NHSS of Jiangsu Province, and the numbers of respondents were 7021, 10,466, and 11,550 in 2008, 2013, and 2018, respectively. Excluding missing data, the total sample in the study contained 25,048 respondents aged over 13. As mentioned above, this sample, spanning 10 years, covers the period of the introduction of full health insurance coverage.

The data from the NHSS includes detailed information about the individuals in households, and the questionnaire included questions about the demographic and socioeconomic characteristics of urban and rural residents, including their sex, marital status, educational level, family size, employment status, income level, self-reported health status, and existing illnesses. The questionnaire also examined urban and rural residents’ healthcare-service needs, utilization, and underutilization, as well as health insurance. Furthermore, it included questions about reasons for not seeking outpatient care (1. Illness is not serious; 2. Financial difficulties; 3. The procedure of seeing a doctor is too complex; 4. Having no time; 5. Transportation problems; 6. No effective treatment; 7. Other), reasons for not seeking inpatient care (1. It is unnecessary; 2. No effective treatment; 3. Financial difficulties; 4. Poor service of hospital; 5. Having no time; 6. There is no bed available; 7. Other), and reasons for early discharge from hospital (1. Cannot recover from illness; 2. Think oneself recovered; 3. Financial difficulties; 4. It costs too much; 5. Poor hospital facilities; 6. Poor medical technology at the hospital; 7. Other). The variables included in the multivariate model are presented in Table 1.

**Table 1:** Descriptions of dependent and independent variables

	Categories	Indicators/survey questions
Dependent variables		
Non-use of outpatient services	=1, Reported non-use of outpatient services =0, No reported non-use of outpatient services	Questions: Have you been ill in the last two weeks? What was the main reason for not seeking outpatient treatment?
Non-use of inpatient services	=1, Reported non-use of inpatient services =0, No reported non-use of inpatient services	Question: In the past year, did a doctor suggest that you needed inpatient care but you were not hospitalized? What was the main reason for not seeking inpatient treatment?
Non-use of physical examinations	=1, Did not undergo physical examination =0, Underwent physical examination	Question: Have you had a physical examination in the past 12 months?
Early discharge from the hospital	=1, Reported early discharge from the hospital =0, No reported early discharge from the hospital	Question: In the year before the survey, were you ever discharged early from hospital? What was the main reason for your self-discharge?
Independent variables		
Predisposing factors		
Sex	= 1, Male, = 2, Female	Question: What is your sex?
Age (years)	=1, if ≤30; =2, 30–45; =3, 45–60; =4, if ≥60	Question: Year of birth.
Marital status	=1, not married;=2, married	Question: What is your marital status? (Widowhood and divorce both belong to “not married”.)
Employment/retirement status	=1, employed; =2, not employed; =3, retired	Question: What is your employment status?
Education level	=1, less than lower secondary; =2, upper secondary & vocational training; =3, tertiary	Question: What is your education level?
Family size	=1, if ≤2 persons in household; =2, if ≥3 persons in household	Number of household members
Enabling factors		
Social health insurance	=1, have insurance; =0, have no insurance	Question: Do you have any social health insurance?
Income level	=1, ≤¥13,334; =2, ¥13,334–24,915; =3, ¥24,915–39,967; =4, ≥¥39,967	Yearly household income divided by the number of household members; first household member with a weight of 1, all following household members with a weight of 0.5.
Area of residence	=1, urban; =2, rural	Question: What is your registered type?
Financial subsidies	=1, financial subsidies from government =2, no financial subsidies from government	Whether the respondent was a Medicaid recipient or in a poor household or low-income household
Need variables		
Self-reported health status(Value on VAS)	=1, excellent (80–100); =2, good (70–80); =3, fair (50–70); =4, poor (≤49)	Question: Which score best represents your health today?
Chronic diseases	=0, no chronic; =1, one chronic; =2, multiple chronic diseases	Questions: Do you have confirmed hypertension? Do you have confirmed diabetes? Do you have other chronic diseases diagnosed?
Depression	=1, depressed; =0, not depressed	Question: What is the extent of your anxiety or depression?
Drinking	=1, yes; =2, no	Whether the respondent ever drank alcoholic beverages or is presently a drinker
Smoking	=1, yes; =2, no	Whether the respondent reports ever having smoked or is presently a smoker

### Statistical analysis

#### Descriptive analysis

Descriptive statistics were used to analyze the sample characteristics, including the prevalence of main reasons for and trends in reported unmet healthcare needs in rural and urban households.

#### Regression analysis

Binary multivariate logistic regression was used to estimate the association between unmet healthcare needs and universal health insurance coverage, along with other factors. Our analysis included a regression of unmet health services and a regression of the main causes of unmet health services. For the first regression, the dependent variable, unmet healthcare need, was defined by “non-use of health services”, which was dichotomized into 0 = “no reported non-use of health services” and 1 = “reported non-use of health services”. For the second regression, the dependent variable “main reason for unmet healthcare need” was defined as follows: “main reason” = 1 and “other reasons” = 0. Comprehensive analyses were conducted, and all data collation and statistical analyses were performed using SPSS 22.0 and Stata 24.0.

### Variable selection

#### Dependent variables

The dependent variables included four kinds of unmet healthcare needs: non-use of outpatient services (a person perceived a need for outpatient care in the past 2 weeks but did not seek outpatient treatment), non-use of inpatient services (a person a perceived a need for inpatient care during the past year but was not hospitalized), non-use of physical examinations (whether or not a person underwent physical examinations in the past year), and early discharge from hospital (whether or not a person was discharged early from hospital in the past year). Furthermore, respondents were asked to specify why they did not seek healthcare treatment. It should be noted that the reasons for physical examinations were not included in the study.

#### Independent variables

Based on the Andersen healthcare utilization model, which has guided systematic investigations into the factors associated with healthcare utilization, we categorized the independent variables into three types: predisposing variables, enabling variables, and need variables [40]. Predisposing factors included demographic characteristics and social structure factors, such as sex, age, marital status, employment status, education level, and family size. Enabling factors included organizational and financial variables that facilitate healthcare utilization [15], consisting of social health insurance, income level, area of residence, and whether financial subsidies are received (in China, the lowest-income households were identified by local governments and subsidized by the local bureau of civil affairs). Taking inflation into account, all income was converted to the price level in 2018 using a price index from China Statistical Yearbook. Need factors refer to both subjective need (self-perceived) and objective need (diagnosed or evaluated by healthcare professionals) for healthcare utilization. The former included self-reported health status, and the latter included smoking, drinking, depression, and chronic diseases. Furthermore, to estimate the effect of the whole process of universal health insurance coverage on unmet healthcare needs, we introduced year dummies and cross-variables of insurance interacting with these year dummies. Detailed descriptions of the samples are included in Table 2.

**Table 2:** Sample characteristics divided by living areas (urban & rural)

	Total	Urban	Rural	*p*
	*n* = 25,048	*n* = 10,973 (43.81%)	*n* = 14,075 (56.19%)	value
Year				0.000
2008	23.95%	12.73%	32.69%	
2013	36.82%	42.61%	32.31%	
2018	39.23%	44.66%	35.00%	
Predisposing factors				
Sex				0.055
Male	48.85%	48.16%	49.39%	
Female	51.15%	51.84%	50.61%	
Age (years)				0.000
≤ 30	17.45%	16.99%	17.80%	
30–45	21.10%	20.39%	21.66%	
45–60	30.32%	26.34%	33.42%	
> 60	31.13%	36.28%	27.11%	
Family size				0.000
≤ 2	25.71%	30.75%	21.79%	
> 2	74.29%	69.25%	78.21%	
Marital status				0.006
Married	80.70%	79.92%	81.31%	
Not married	19.30%	20.08%	18.69%	
Education level				0.000
Less than lower secondary	68.63%	53.71%	80.26%	
Upper secondary & vocational training	18.36%	23.48%	14.37%	
Tertiary	13.01%	22.81%	5.37%	
Employment status				0.000
Employed	64.94%	48.55%	77.71%	
Not employed	19.53%	19.02%	19.94%	
Retired	15.53%	32.43%	2.35%	
Enabling factors				
Income level				0.000
≤ ¥13,334	25.44%	8.38%	38.74%	
¥13,334–24,915	24.60%	19.45%	28.61%	
¥24,915–39,967	25.10%	32.39%	19.42%	
> ¥39,967	24.86%	39.78%	13.23%	
Financial subsidies				0.000
Yes	3.78%	2.83%	4.53%	
No	96.22%	97.17%	95.47%	
Social health insurance				0.000
Yes	96.47%	96.14%	96.74%	
No	3.53%	3.86%	3.26%	
Need factors				
Depression				0.000
Not depressed	94.83%	96.26%	93.71%	
Depressed	5.17%	3.74%	6.29%	
Self-reported health status (value on VAS)				0.000
Excellent (80–100)	75.95%	75.19%	76.53%	
Good (70–80)	12.84%	14.01%	11.93%	
Fair (50–70)	9.78%	9.63%	9.90%	
Poor (< 50)	1.43%	1.17%	1.64%	
Smoking				0.056
Yes	26.05%	25.45%	26.52%	
No	73.95%	74.55%	73.48%	
Drinking				0.024
Yes	22.67%	21.99%	23.20%	
No	77.33%	78.01%	76.80%	
Chronic diseases				0.000
0	69.06%	63.56%	73.34%	
One	23.55%	26.60%	21.17%	
Multiple	7.39%	9.84%	5.49%	

## Results

### Sample characteristics

As shown in Table 2, among all respondents in Jiangsu province, 43.81% (10973) were urban residents and 56.19% (14075) were rural residents, and 23.95, 36.82, and 39.23% were from the NHSS in 2008, 2013, and 2018, respectively. More than two-thirds of people (74.29%) had a larger family size (> 2). People with lower education levels (less than secondary) in rural areas accounted for 80.26%, which was higher than in urban areas (53.71%). In urban areas, almost half of residents were retired or unemployed, compared with approximately 21% in rural areas. There were more people in the highest income group (> 39,967) in urban areas (39.78%) than in rural areas (13.23%). The insurance coverage rate exceeded 95% in both urban and rural areas. Of which, 11.87% of the respondents participated in URMI, and 28.55% in NRCM. The proportion of people who were depressed was 5.17%. The proportions of people whose self-reported health status was excellent were 75.19 and 76.53% in urban and rural areas, respectively. The proportion of people with one or multiple chronic diseases was higher in urban areas (26.60 and 9.84%) than in rural areas (21.17 and 5.49%).

### Prevalence of, reasons for, and trends in unmet healthcare needs

**Fig. 1:**
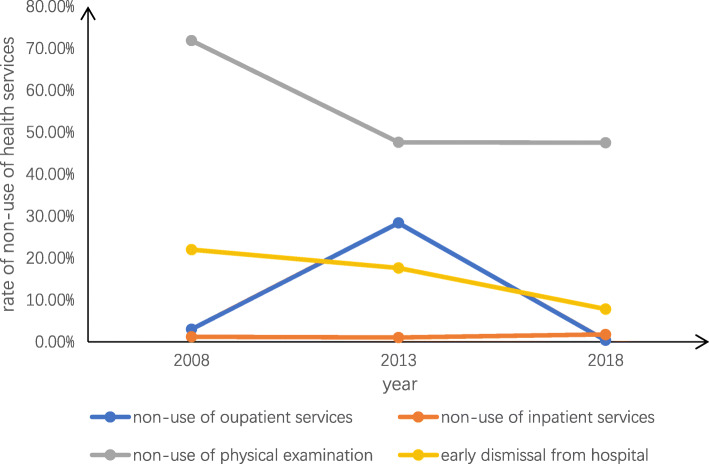
Trends in overall unmet healthcare needs

**Fig. 2:**
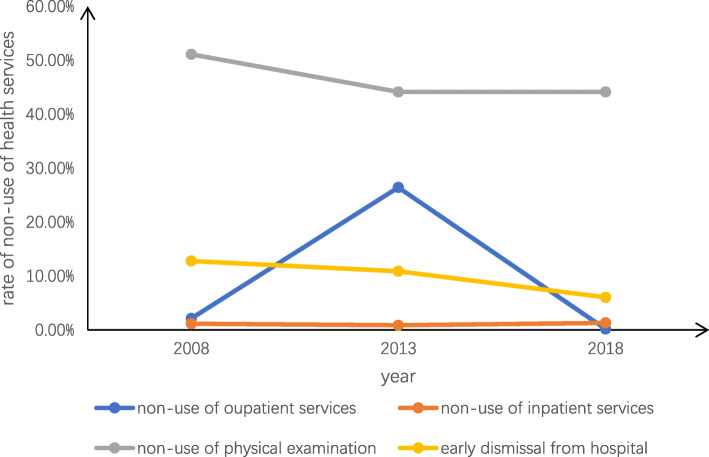
Trends in urban unmet healthcare needs

**Fig. 3:**
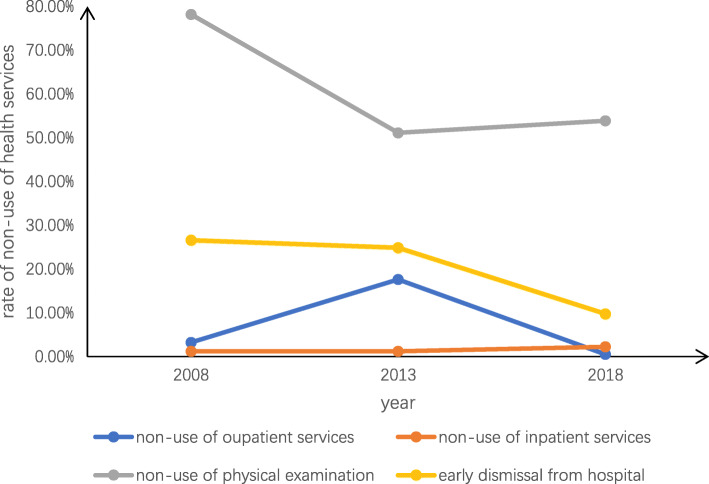
Trends in rural unmet healthcare needs

Table 3 details the prevalence of and the reasons for unmet healthcare needs in Jiangsu province. The trends in unmet healthcare needs from 2008 to 2018 are also shown in Figs. 1, 2, 3. Overall, the prevalence of non-use of outpatient services was 8.99%, which was higher in urban areas (11.63%) than in rural areas (6.93%). The overall main reason for not seeking outpatient services was “Illness is not serious” (11.68%), and this was also true in urban areas (7.94%). However, in rural areas, the main reason was “No effective treatment” (44.84%). The total prevalence of non-use of inpatient services and physical examinations were 1.37 and 53.37%, respectively. The corresponding figures were higher among people in rural areas than those in urban areas. The overall primary reason for not seeking inpatient services was “Financial difficulties” (35.73%), and this was also the main reason in rural areas (41.41%); however, in urban areas, “It is unnecessary” was the main reason (39.17%). In addition, 13.16% of respondents left the hospital before recovery. As a whole, the main reason for this was “Think oneself recovered” (27.92%), and this was also true in urban areas (39.76%). However, in rural areas the leading reason for early discharge from hospital was “Other reasons” (25.27%), followed by “Financial difficulties” (24.73%).

**Table 3:** Prevalence and reasons of unmet healthcare needs

	total	urban	rural	P值
**Non-use of outpatient services**	8.99%(2252)	11.63%(1276)	6.93%(976)	0.000
2008	3.00%	2.15%	3.26%	
2013	28.35%	26.43%	17.62%	
2018	0.36%	0.20%	0.51%	
**Reasons for not seeking outpatient services**				0.000
Illness is not serious	11.68%	7.94%	16.56%	
Financial difficulties	2.76%	0.79%	5.35%	
The procedure of seeing a doctor is complex	0.40%	0.39%	0.41%	
Having no time	0.76%	0.16%	1.34%	
Transportation problems	0.09%	0.08%	0.10%	
No effective treatment	2.41%	0.55%	44.84%	
Other	82.00%	90.09%	71.40%	
**Non-use of inpatient services**	1.37%(343)	1.10%(121)	1.58%(222)	0.001
2008	1.20%	1.15%	1.22%	
2013	1.05%	0.88%	1.23%	
2018	1.77%	1.31%	2.23%	
**Reasons for not seeking inpatient services**				0.000
It is unnecessary	27.38%	39.17%	21.15%	
No effective treatment	4.32%	5.00%	3.96%	
Financial difficulties	35.73%	25.00%	41.41%	
Poor service of hospital	0.86%	1.67%	0.44%	
Having no time	15.56%	5.83%	20.70%	
There is no bed available	2.59%	5.83%	0.88%	
Other	13.54%	17.50%	11.45%	
**Non-use of physical examination**	53.37%(13,369)	43.69%(4794)	60.92%(8575)	0.000
2008	71.87%	51.11%	78.18%	
2013	47.59%	44.14%	51.13%	
2018	47.51%	44.14%	53.85%	
**Early discharge from hospital**	13.16%(268)	8.43%(85)	17.80%(183)	0.000
2008	22.01%	12.79%	26.59%	
2013	17.65%	10.88%	24.86%	
2018	7.84%	6.06%	9.78%	
**Reasons for Early discharge from hospital**				0.000
Cannot recover from illness	10.19%	13.25%	8.79%	
Think oneself recovered	27.92%	39.76%	22.53%	
Financial difficulties	20.00%	9.64%	24.73%	
Cost too much	12.45%	9.64%	13.74%	
Poor hospital facilities	1.51%	0.00%	2.20%	
Poor medical technology at the hospital	3.02%	3.61%	2.75%	
Other	24.91%	24.10%	25.27%

With regard to the trend in unmet healthcare needs, on the whole, the underutilization rates in urban and rural areas were similar. The overall rates of non-use of outpatient services (2008:3.00%; 2018:0.36%), non-use of physical examinations (2008:71.87%; 2018:47.51%), and early discharge from hospital (2008:22.01%; 2018: 7.84%) all showed decreasing trends. However, in 2013, the rate of non-use of outpatient services (2013:28.35%) showed a dramatic rise, followed by a significant decline from 2013 to 2018. For non-use of inpatient services, the rate remained relatively lower and was flat from 2008 to 2018(2008:1.20%; 2013:1.05%; 2018:1.77%). Furthermore, the range of fluctuation in the rate of non-use of outpatient services was greater in in rural areas(2008:3.26%; 2018:0.51%) than urban areas(2008:2.15%; 2018:0.2%). The rate of non-use of physical examinations demonstrated a greater decline in rural areas(2008:78.18%; 2013:51.13%%) than in urban areas(2008:51.11%; 2013:44.14%) from 2008 to 2013. In addition, the decline in the rate of early discharge from hospital was more significant in rural areas(2008:26.59%; 2018:9.18%) than in urban areas(2008:12.79%; 2018:6.06%) from 2008 to 2018.

### Multivariate regression results

#### Logistic regression of unmet healthcare needs

The data shown in Table 4 indicate that people aged over 60 demonstrated a significantly higher likelihood of reporting non-use of outpatient services (OR = 2.045), non-use of inpatient services (OR = 1.672), and early discharge from hospital (OR = 2.187). However, the correlation was opposite in the non-use of physical examinations (OR = 0.337). Those who had received tertiary education were significantly less likely to report underutilization of all kinds of healthcare services. Unemployed or retired people demonstrated a higher probability of non-use of inpatient services (retired: OR = 1.373) and physical examinations (unemployed: OR = 1.507). Those living in larger families (households > 2 people) were significantly less likely to report outpatient (OR = 0.954) and inpatient underutilization (OR = 0.905).

**Table 4:** Logistic regression of unmet healthcare needs among the whole population

	Non-use of outpatient services		Non-use of inpatient services		Non-use of physical examination		Early discharge from hospital	
	OR	SD	OR	SD	OR	SD	OR	SD
**Predisposing factors**								
Sex, ref.: male	1.283	0.193	1.210	0.498	1.125	0.181	1.156	0.191
Age (years), ref.:≤30								
30–45	1.705***	0.258	1.006	0.745	0.909*	0.051	1.110	0.491
45–60	2.451***	0.153	1.465***	0.309	0.617*	0.139	1.906	0.874
> 60	2.045***	0.359	1.672**	0.295	0.337***	0.024	2.187*	1.188
Marital status, ref.: not married	1.293	0.288	1.408	0.507	1.920	0.086	1.047	0.339
Education level, ref.: less than lower secondary								
Upper secondary & vocational training	1.287	0.404	0.788	0.453	0.763***	0.048	0.907*	0.276
Tertiary	0.693*	0.218	0.457***	0.121	0.392**	0.111	0.521**	0.104
Employment status, ref.: employment								
Unemployment	1.406	0.339	1.266	0.581	1.507*	0.431	1.751	1.157
Retired	1.375	0.239	1.373**	0.208	1.298	0.209	1.443	0.998
Family size, ref.: ≤2	0.954*	0.168	0.905**	0.176	1.030	0.200	0.774	0.259
**Enabling factors**								
Social health insurance, ref.: have no insurance	0.492***	0.094	0.586**	0.097	1.251	0.417	0.943*	0.327
Income level, ref.: ≤¥13,334								
¥13,334–24,915	1.210	0.309	0.804	0.313	0.902***	0.011	1.094	0.469
¥24,915–39,967	0.902**	0.183	0.512***	0.092	0.496***	0.020	1.336	0.764
> ¥39,967	0.783*	0.093	0.483**	0.102	0.410***	0.030	0.689**	0.244
Financial subsidies: ref.: have financial subsidies	0.695	0.288	0.581*	0.131	0.958	0.141	0.904*	0.180
Area of residence, ref.: urban	1.417***	0.093	1.408**	0.111	1.573*	0.057	2.804**	0.571
**Need factors**								
Self-reported health status (value on VAS), ref.: excellent								
Good	1.223	0.223	1.435**	0.217	1.032	0.061	1.508	0.991
Fair	0.931	0.147	1.207	0.413	1.370	0.099	1.349	0.409
Poor	1.309**	0.109	2.509**	0.509	1.234	0.155	2.097	1.518
Depression, ref.: not depressed	1.437**	0.200	1.234	0.495	1.003**	0.063	1.093*	0.209
Smoking, ref.: smoked	1.237	0.313	1.496	0.190	0.926	0.095	0.682	0.209
Drinking, ref.: drank	0.601*	0.134	0.957	0.310	1.008	0.058	1.588	0.970
Chronic disease, ref.: 0								
One chronic disease	1.985***	0.433	1.973**	0.259	0.744***	0.038	1.210	0.506
Multiple chronic diseases	3.063***	0.908	2.522*	0.648	0.253**	0.021	1.606	0.974
Year, ref.: 2008								
2013	0.861***	1.032	0.909	0.280	0.773**	0.148	1.107	0.579
2018	0.603***	0.243	0.547**	0.109	0.581*	0.230	0.487*	0.105
Year2013* Social health insurance	0.609**	0.083	1.064	0.896	0.896	0.287	0.541*	0.078
Year2018* Social health insurance	0.235**	0.131	0.432**	0.090	0.609	0.172	0.309**	0.091

Note: *** indicates *p* value < 0.001, ** indicates *p* value < 0.01, * indicates *p* value < 0.05, *SD* standard deviation, *OR* odd ratio

Regarding enabling factors, we found that having health insurance was correlated with a significantly lower probability of non-use of outpatient (OR = 0.492) and inpatient (OR = 0.586) services and early discharge from hospital (OR = 0.943). The highest income group was significantly less likely to report healthcare underutilization. In addition, receiving financial subsidies was correlated with a significantly lower likelihood of inpatient underutilization (OR = 0.581) and early discharge from hospital (OR = 0.904). Rural residents were significantly more likely to report non-use of health services than urban residents. Among need factors, the likelihood of underutilization of outpatient (OR = 1.309) and inpatient services (OR = 2.509) was significantly higher among people with a poor health status. The depressed demonstrated a higher likelihood of healthcare underutilization. People with one or multiple chronic diseases had a significantly higher probability of non-use of outpatient services (OR = 1.985, 3.063) and inpatient services (OR = 1.973, 2.522), but showed a lower likelihood of underutilization of physical examinations (OR = 0.744, 0.253).

The probability of non-use of outpatient services and physical examinations was significantly lower both in 2013 (OR = 0.861, 0.773) and 2018 (OR = 0.603, 0.581) than in 2008, and the corresponding odds ratios declined from 2013 to 2018. In addition, the likelihoods of non-use of inpatient services (OR = 0.547) and early discharge from hospital (0.487) in 2018 were significantly lower than in 2008. According to the coefficient, people with health insurance both in 2013 and 2018 demonstrated a significantly lower likelihood of reporting non-use of outpatient services and early discharge from hospital, while the corresponding figures were both smaller in 2018 (OR = 0.235, 0.309) than in 2013 (OR = 0.609, 0.541). Additionally, individuals having health insurance in 2018 were also significantly less likely to report non-use of inpatient services (OR = 0.432).

Tables 5 and 6 show logistic regressions of the non-use of health services among people living in urban and rural areas, respectively. The married demonstrated a significantly lower likelihood of non-use of outpatient services (OR = 0.749) and physical examinations (OR = 0.801), and a higher likelihood of non-use of inpatient services (OR = 1.177) in urban areas, but there was no significant effect in rural areas. In addition, the positive effect of education level on healthcare utilization was estimated to be greater among urban residents, while the mitigating effect of a higher income level on unmet healthcare needs was greater among rural residents. The effect of health insurance on reducing the likelihood of hospitalization underutilization and early discharge was estimated to be greater in rural areas (OR = 0.291, 0.628) than in urban areas (OR = 0.610, 0.793). The likelihood of non-use of health services was significantly lower in 2018 in both rural and urban areas, but the year effect was estimated to be greater in rural areas. People having health insurance in 2018 demonstrated a significantly lower likelihood of non-use of outpatient services (OR = 0.689) and physical examinations (OR = 0.947) in urban areas, while individuals in rural areas with health insurance both in 2013 and 2018 were significantly less likely to report non-use of outpatient (2013: OR = 0.847, 2018: OR = 0.493) and inpatient services (2013: OR = 0.904, 2018: OR = 0.312). In addition, people having health insurance in 2018 were significantly less likely to report early discharge from hospital (OR = 0.271) in rural areas, but there was no significant effect in urban areas.

**Table 5:** Logistic regression of unmet healthcare needs among people living in urban areas

	Non-use of outpatient services		Non-use of inpatient services		Non-use of physical examination		Early discharge from hospital	
	OR	SD	OR	SD	OR	SD	OR	SD
**Predisposing factors**								
Sex, ref.: male	1.148	0.427	0.829	0.391	0.892	0.532	0.804	0.290
Age (years), ref.: ≤30								
30–45	1.709	0.743	1.096	0.413	0.934	0.561	2.484	1.509
45–60	1.937**	0.418	1.579	0.735	0.839**	0.211	2.299	1.280
> 60	1.897***	0.502	1.227**	0.186	0.320***	0.094	1.927	1.142
Marital status, ref.: not married	0.749*	0.158	1.177*	0.230	0.801**	0.227	0.876	0.298
Education level, ref.: less than lower secondary								
Upper secondary & vocational training	0.902	0.416	0.901**	0.187	0.810***	0.235	0.892*	0.208
Tertiary	0.528**	0.201	0.473***	0.104	0.407***	0.103	0.533*	0.118
Employment status, ref.: employment								
Unemployment	1.311**	0.198	1.307***	0.311	1.630*	0.413	1.191	0.413
Retired	1.505	0.479	1.028	0.279	1.419***	0.211	1.485	0.290
Family size, ref.:≤2	0.884	0.517	0.955	0.618	0.997	0.359	0.908	0.305
**Enabling factors**								
Social health insurance, ref.: have no social medical health insurance	0.637**	0.210	0.610**	0.204	0.953*	0.249	0.793**	0.238
Income level, ref.: ≤¥13,334								
¥13,334–24,915	0.811	0.167	0.807	0.492	0.992	0.475	1.138	0.523
¥24,915–39,967	0.774	0.225	0.906	0.298	0.758	0.343	0.872	0.503
> ¥39,967	0.804**	0.174	0.588**	0.104	0.479***	0.213	0.849*	0.176
Financial subsidies: ref.: have financial subsidies	1.100	0.590	0.905*	0.228	1.381	0.509	0.871*	0.203
**Need factors**								
Self-reported health status (value on VAS), ref.: excellent								
Good	1.588	0.530	1.843	0.998	1.227	0.589	1.408	0.690
Fair	0.990	0.491	1.266	0.680	1.183	0.391	1.249	0.754
Poor	1.098***	0.199	1.320*	0.388	1.909	0.998	1.907	0.904
Depression, ref.: not depressed	1.554**	0.379	1.579**	0.193	1.202*	0.236	1.190*	0.183
Smoking, ref.: smoked	1.073	0.405	1.379	0.590	0.838	0.455	1.093	0.158
Drinking, ref.: drank	0.805**	0.217	0.821*	0.303	1.384	0.849	0.953	0.418
Chronic disease, ref.: 0								
One chronic disease	1.326***	0.126	2.075**	0.310	0.522***	0.109	0.973	0.381
Multiple chronic disease	1.182**	0.321	1.912***	0.274	0.256***	0.071	1.278	0.671
Year, ref.:2008								
2013	0.891	0.391	0.933	0.438	0.917**	0.208	0.923	0.293
2018	0.502*	0.179	0.827	0.311	0.718**	0.124	0.809**	0.243
Year2013*Social health insurance	0.722	0.439	1.079	0.591	1.145	0.516	0.917	0.448
Year2018*Social health insurance	0.689**	0.173	0.805	0.388	0.947*	0.207	0.675	0.289

Note: *** indicates *p* value < 0.001, ** indicates *p* value < 0.01, * indicates *p* value < 0.05, *SD* standard deviation, *OR* odd ratio

**Table 6:** Logistic regression of unmet healthcare needs among people living in rural areas

	Non-use of outpatient services		Non-use of inpatient services		Non-use of physical examination		Early discharge from hospital	
	OR	SD	OR	SD	OR	SD	OR	SD
**Predisposing factors**								
Sex, ref.: male	1.447	0.293	1.191	0.610	0.822**	0.119	1.384	0.475
Age (years), ref.: ≤30								
30–45	1.822*	0.410	1.906	1.238	0.815	0.260	1.191	0.549
45–69	2.022***	0.605	1.508*	0.709	0.944*	0.128	1.527**	0.318
> 60	2.833**	0.874	2.193**	0.944	0.410**	0.091	2.299**	0.904
Marital status, ref.: not married	1.407	0.830	1.310	0.855	1.380	0.473	1.138	0.710
Education level, ref.: less than lower secondary								
Upper secondary & vocational training	1.190	0.268	1.105	0.488	0.699***	0.102	1.135	0.487
Tertiary	1.038	0.409	0.653**	0.179	0.544***	0.099	0.909*	0.232
Employment status, ref.: employment								
Unemployment	0.984	0.191	1.470*	0.333	1.903*	0.336	1.705	0.933
Retired	1.122	0.287	1.277*	0.184	1.190**	0.181	1.134	0.574
Family size, ref.: ≤2	0.810*	0.180	0.739***	0.210	1.163	0.719	1.209	0.484
**Enabling factors**								
Social health insurance, ref.: have no social medical health insurance	0.770**	0.218	0.291***	0.098	1.372	0.388	0.628**	0.134
Income level, ref.: ≤¥13,334								
¥13,334–24,915	1.237	0.575	0.803*	0.193	0.788	0.238	0.871**	0.214
¥24,915–39,967	0.717*	0.129	1.024	0.436	0.610**	0.131	0.910*	0.165
> ¥39,967	0.579**	0.100	0.337**	0.079	0.427**	0.091	0.762**	0.104
Financial subsidies: ref.: have financial subsidies	0.474	0.187	0.657**	0.184	0.983	0.318	0.904	0.354
**Need factors**								
Self-reported health status (value on VAS), ref.: excellent								
Good	1.008	0.198	1.390	0.550	0.974	0.449	0.790*	0.111
Fair	1.478*	0.311	1.905**	0.338	1.135	0.681	1.353	0.381
Poor	1.809*	0.284	2.497***	0.682	1.036	0.382	0.899	0.401
Depression, ref.: not depressed	1.198	0.410	1.409	0.549	1.210**	0.229	1.137*	0.204
Smoking, ref.: smoked	1.471	0.625	1.191	0.781	0.997***	0.148	1.241	0.458
Drinking, ref.: drank	1.191	0.406	0.903	0.359	0.974	0.402	0.977	0.319
Chronic disease, ref.:0								
One chronic disease	2.871**	0.792	3.040*	0.939	0.609**	0.103	1.228	0.514
Multiple chronic disease	4.190***	0.690	6.093***	1.109	0.426***	0.074	1.033*	0.201
Year, ref.: 2008								
2013	0.917**	0.209	1.099	0.824	0.737	0.198	1.036	0.578
2018	0.362**	0.087	0.634**	0.162	0.608***	0.130	0.638**	0.201
Year2013*Social health insurance	0.847*	0.219	0.904**	0.289	0.610	0.248	0.931	0.220
Year2018*Social health insurance	0.493**	0.170	0.312**	0.108	0.484	0.150	0.271**	0.083

Note: *** indicates *p* value < 0.001, ** indicates *p* value < 0.01, * indicates *p* value < 0.05, *SD* standard deviation, *OR* odd ratio

#### Logistic regression of main reasons for unmet healthcare needs

The results of the regression of unmet healthcare needs caused by a single main reason are shown in Table 7. The most frequently reported reasons for not seeking outpatient care, inpatient care, and early discharge from hospital were “Illness is not serious”, “Financial difficulties”, and “Think oneself recovered”, respectively. We found that females (OR = 1.576) and those aged over 45 (45–60: OR = 1.392; > 60: OR = 1.838) were significantly more likely to report underutilization of outpatient services for the reason “Illness is not serious”. Individuals with higher education, who were retired, or had one or more chronic diseases demonstrated a significantly lower likelihood of non-use of outpatient services due to “Illness is not serious”. Compared with individuals in 2008, those in 2018 were significantly less likely to report non-use of outpatient services (OR = 0.954) due to the cause “Illness is not serious”.

**Table 7:** Logistic regression of unmet healthcare needs caused by main reasons

	Non-use of outpatient services		Non-use of inpatient services		Early discharge from hospital	
	OR	SD	OR	SD	OR	SD
**Predisposing factors**						
Sex, ref.: male	1.576*	0.314	0.736	0.304	1.185**	0.190
Age (years), ref.: ≤30						
30–45	1.415	0.782	1.578	0.908	1.406	0.903
45–60	1.392**	0.248	1.389	0.601	1.876**	0.628
> 60	1.838**	0.178	1.739**	0.505	2.171**	0.918
Marital status, ref.: not married	0.949	0.370	1.313	0.790	1.375*	0.291
Education level, ref.: less than lower secondary						
Upper secondary & vocational training	0.829*	0.247	0.709	0.353	0.945	0.400
Tertiary	0.597**	0.186	0.874**	0.227	0.639**	0.194
Employment status, ref.: employment						
Unemployment	0.997	0.558	1.297**	0.274	0.948	0.470
Retired	0.495***	0.097	1.507	0.622	1.193	0.593
Family size, ref.: ≤2	1.190	0.546	0.903*	0.114	1.354	0.742
**Enabling factors**						
Social health insurance, ref.: have no insurance	1.275	0.336	0.312***	0.083	0.875	0.501
Income level, ref.: ≤¥13,334						
¥13,334–24,915	0.974	0.483	0.629**	0.148	1.094	0.590
¥24,915–39,967	0.850	0.324	0.310***	0.077	0.905	0.532
> ¥39,967	0.645	0.290	0.202***	0.082	0.654*	0.212
Financial subsidies: ref.: have financial subsidies	1.290	0.596	0.387***	0.115	1.099	0.601
Area of residence, ref.: urban	0.840	0.250	1.179	0.596	1.178	0.578
**Need factors**						
Self-reported health status (value on VAS), ref.: excellent						
Good	1.180	0.623	1.820	0.793	0.879	0.283
Fair	0.908	0.421	1.576	0.669	0.913*	0.301
Poor	0.718	0.207	1.845	0.858	0.698	0.411
Depression, ref.: not depressed	0.747**	0.190	1.094	0.480	0.855**	0.146
Smoking, ref.: smoked	1.009	0.393	1.003	0.502	1.084	0.394
Drinking, ref.: drank	0.890	0.155	0.822	0.348	0.747	0.401
Chronic disease, ref.:0						
One chronic disease	0.735***	0.110	1.997**	0.839	0.693*	0.171
Multiple chronic disease	0.379***	0.130	2.624**	1.095	0.402**	0.103
Year, ref.:2008						
2013	1.090	0.388	0.897*	0.248	1.403	0.834
2018	0.954*	0.173	0625**	0.173	0.811	0.338
Year2013*Social health insurance	1.032	0.491	0.901	0.418	1.193	0.579
Year2018*Social health insurance	0.832	0.290	0.309**	0.104	0.975	0.628

Note: *** indicates *p* value < 0.001, ** indicates *p* value < 0.01, * indicates *p* value < 0.05, *SD* standard deviation, *OR* odd ratio

Individuals aged over 60 (OR = 1.739) or who were unemployed (OR = 1.297) demonstrated a significantly higher likelihood of underutilization of inpatient services due to “Financial difficulties”, while those who had received tertiary education (OR = 0.874) and those living in larger families (> 2: OR = 0.903) exhibited a significantly lower probability of underutilization of inpatient services. Having insurance or being in a higher income group were correlated with a significantly lower likelihood of inpatient underutilization due to “Financial difficulties”. In addition, people having chronic diseases were significantly more likely to report non-use of inpatient services resulting from financial problems (one: OR = 1.997; multiple: OR = 2.624). Respondents in 2018 and 2013 were significantly less likely to report inpatient underutilization resulting from financial issues in comparison with individuals in 2008, and the odds ratio was estimated to be lower in 2018. Moreover, people having health insurance in 2018 also indicated a significantly lower likelihood of non-use of inpatient services (OR = 0.309) due to “Financial difficulties”. Those who were female (OR = 1.185), aged over 60 (OR = 2.171), or unmarried (OR = 1.375) were more likely to report early discharge from hospital due to the cause “Think oneself recovered”. Meanwhile, individuals having a tertiary education (OR = 0.639), in the highest income group (OR = 0.654), or having chronic disease demonstrated a significantly lower probability of early discharge from hospital resulting from thinking themselves recovered.

## Discussion

Reducing unmet healthcare needs is very important for alleviating inequality in health services and achieving universal health coverage [22]. Although many studies have explored healthcare utilization and underutilization, little research has examined the association between universal health insurance coverage and unmet healthcare needs. In this study, we analyzed the prevalence of, main reasons for, and trends in unmet healthcare needs over the period of the introduction of universal health insurance coverage in China and make comparisons between urban and rural areas. Additionally, we estimated the effects of universal health insurance coverage and other socioeconomic factors on unmet healthcare needs.

### Prevalence, main reasons and trends of unmet healthcare needs

On the whole, the degrees of reported non-use of outpatient services, inpatient services, physical examinations, and early discharge from hospital in Jiangsu Province were significantly different. The prevalence of non-use of physical examinations was much higher than other kinds of healthcare services, which may be due to physical examinations not being included in China’s health insurance system and people lacking awareness of preventive healthcare. Hence, policy efforts should focus on further expanding the service package of health insurance and adopting strategies to improve the effective utilization of preventive healthcare [12].

The prevalence of unmet healthcare needs in rural areas was found to be generally higher than that in urban areas, which is in line with previous surveys [22, 30, 31, 33]. The main reason for outpatient underutilization overall and in urban areas was “Illness is not serious”, while in rural areas it was “No effective treatment”, a discrepancy that may be caused by high-quality medical resources tending to be allocated to urban areas in China [41]. Therefore, narrowing the gap in allocation of health resources between urban and rural areas should be an effective way to alleviate unmet outpatient needs. The prevalence of non-use of inpatient services and physical examinations was higher among people in rural areas than in urban areas, and financial problems were always the main obstacle to rural residents using inpatient services. This finding reflects the huge economic discrepancy between urban and rural areas in China.

Aside from the non-use of inpatient services, the trends in unmet healthcare needs in urban and rural areas were similar, showing a decline from 2008 to 2018, across the period of implementation of universal health insurance coverage in China. The rate of non-use of inpatient services remained relatively lower and was flat from 2008 to 2018, revealing that, in addition to economic factors, the severity of a person’s disease may mainly determine their choice of inpatient services. Therefore, the rate of non-use of inpatient services remained low and stable. It should be noted that the outpatient underutilization rate showed a dramatic rise from 2008 to 2013, followed by a significant decline from 2013 to 2018. In the early stage of the New Medical Reform Plan in 2009, China’s health insurance coverage rate increased rapidly, but the reimbursement ratio remained low, meaning unmet outpatient service needs continued to increase. The rate of unmet outpatient service needs may have started to decline after 2013 as a result of improvement in the reimbursement level of the health insurance system.

### Association between insurance and its universal coverage and unmet healthcare needs

Health insurance had a significant impact on the reduction of health service underutilization, except for in the case of physical examinations services. With regard to the annual effect, the probability of non-use of outpatient services and physical examinations significantly declined both in 2013 and 2018, and the likelihood of non-use of inpatient services and early discharge from hospital also significantly reduced in 2018. This finding indicates that unmet healthcare needs were alleviated with the development of the economy and deepening medical reform in China. People having health insurance in 2013 and 2018 demonstrated a significantly lower likelihood of non-use of outpatient services and early discharge from hospital, and the odds ratios decreased over time. This finding reflects a continuously positive role of universal health insurance coverage on reducing unmet healthcare needs. However, the effect on non-use of inpatient services was relatively weak.

In addition, the effect of health insurance and its universal coverage on reducing unmet healthcare needs was estimated to be greater in rural than in urban areas. Therefore, universal health insurance coverage played a greater role in promoting effective health utilization in rural areas [42]. Since the prevalence of unmet healthcare needs in rural areas was higher than that in urban areas, policies should focus on further improving the health insurance system for rural residents, for example by achieving full coverage, gradually increasing the reimbursement level and expanding the service package of health insurance, to alleviate the inequality in healthcare utilization between urban and rural areas.

Universal health insurance coverage only significantly affected non-use of inpatient services caused by financial difficulties. This is mainly due to the fact that health insurance is an important economic guarantee for inpatients. The effect of health insurance on reducing the non-use of inpatient services was significant in 2018, indicating that the reform and adjustment of health insurance in recent years, such as the policy of critical illnesses payment and extending the national medicine catalog of medical insurance, may ease the economic burden on patients and promote the utilization of hospitalization.

### Other associated factors of unmet healthcare needs

Other demographic or socioeconomic factors associated with unmet healthcare needs included age, educational level, income level, area of residence, self-reported health status, depression, and chronic diseases. Specifically, older people were significantly less likely to report underutilization of physical examinations [43, 44]. The prevalence of chronic diseases and resultant disabilities was higher among the elderly, and this may lead to a greater need for physical examinations [45]. However, the association between the other three kinds of unmet healthcare needs and age was found to be the opposite, which is in contrast to other reports [12, 14, 46, 47]. Higher education was associated with lower prevalence of non-use of healthcare services, which is in line with work by Li et al. [48]. Therefore, the government could promote residents’ health awareness by improving education, and this may boost the effective utilization of healthcare. The unemployed or retired were more likely to face barriers in obtaining needed health services [12, 13, 19, 48]. Compared with urban residents, rural residents who were unemployed, retired, or who had lower income demonstrated a higher likelihood of having unmet healthcare needs, especially inpatient services. Therefore, strategies such as increasing financial support, widening employment channels in rural areas, and adjusting the urban and rural income distribution system should be implemented to promote healthcare utilization among these people.

Consistent with existing reports [45, 46, 49–52], the overall likelihood of non-use of outpatient and inpatient services was significantly higher among those with lower self-reported health status, who were depressed, or who had chronic diseases. This may be explained by the fact that people who are depressed or have chronic diseases are generally accompanied by long-term or lifelong treatment and medication, which will certainly result in them having more unmet healthcare needs than healthy individuals, especially if there is a lack of patient compliance [12]. However, having chronic diseases was significantly associated with lower underutilization of physical examinations, indicating that chronic patients may have a stronger awareness of disease prevention [12, 15].

Some limitations of our study must be acknowledged. First, unmet healthcare needs in this paper only refer to people who perceived a need for healthcare but did not seek treatment; those who did not perceive a need for healthcare were not included. Therefore, the prevalence of unmet healthcare needs might have been underestimated to some extent. Also, we did not consider the moral hazard which might overestimate the likelihood of unmet healthcare needs to some extent. In addition, partial voluntariness of URMI might lead to adverse selection and affect the results to a certain extent. Finally, the data in our study was from the Jiangsu Province data in the NHSS, which may not be nationally representative.

## Conclusions

Our findings suggest that health insurance significantly reduced unmet healthcare needs in Jiangsu Province. People having health insurance in 2013 and 2018 demonstrated a significantly lower likelihood of non-use of outpatient services and early discharge from hospital, and the odds ratios decreased over time. In addition, the prevalence of unmet healthcare needs in rural areas was found to be higher than that in urban areas, meanwhile universal health insurance coverage played a greater role in reducing unmet healthcare needs in rural areas. Therefore, achieving full coverage and gradually increasing the reimbursement level and expanding the service package of health insurance should be an effective way to promote effective health utilization in rural areas and alleviate the inequality in healthcare utilization between urban and rural areas. Other socioeconomic factors, such as age, marital status, educational level, employment status, income level, and health status, were found to significantly affect unmet healthcare needs. Therefore, policy efforts need to address these factors to promote effective healthcare utilization by residents.

## Data Availability

The datasets used in the current study are not publicly available due to the confidential policy but are available from the corresponding author on reasonable request.
